# American Institute of Homœopathy

**Published:** 1892-04

**Authors:** Pemberton Dudley

**Affiliations:** General Secretary; Fifteenth and Master Streets, Philadelphia, Pa.


### AMERICAN INSTITUTE OF HOMŒOPATHY.

### SECRETARY'S NOTICE OF SESSION OF 1892.

The Annual Session of the Institute for 1892 will be held in Cornwall's Hall, Washington, D. C., beginning on Monday afternoon, June 13th, and continuing until Friday, June 17th. (See Transactions of 1890, page 63.) Monday afternoon will be devoted to preliminary and routine business, and in the evening the President's Address will be delivered and the Memorial Service held.

The proprietors of Willard's Hotel, the Ebbitt House, and the Rigg's House have contracted with the Local Committee of Arrangements to accommodate physicians and friends accompanying them at the uniform rate of three dollars per day. The Local Committee will establish its headquarters at Willard's, and they request and advise that all engagements of rooms at any of these houses be made through their Chairman, Dr. J. B. G. Custis, or their Secretary, Dr. Wm. R. Ring.

The preparatory work of the Bureau is being prosecuted with more than usual energy, with special efforts to secure an intelligent and profitable discussion of the papers. Essayists, who wish their papers well discussed, should place duplicate copies in the hands of the appropriate Chairman at least one month prior to the meeting.

The Session of 1892 presents some special claims to the support of all homœopathic physicians. To keep alive the prestige and influence gained at the meeting of the International Congress; to encourage the growth of Homœopathy in the Southern States; to present a strong front to the governmental officials assembled at Washington; to antagonize the schemes now taking shape for the subversion of professional liberty among the physicians practicing in and around our National Capital; to take action respecting the boycotting of homœopathic physicians by life insurance companies; to further increase the numerical strength and influence of our national society, and to prepare for a proper display of our power and importance as a profession to the peoples who will visit our shores during the Columbian Exposition—these are some of the motives and objects that should determine and secure a very large and enthusiastic meeting of the Institute at Washington next June.

The Secretary's Annual Circular, to be issued in May, will contain information concerning railroad rates and facilities, and a complete programme of the business of the session. Any physician failing to receive a copy, can obtain it on application. Membership in the Institute is open to all homœopathic physicians in good standing. A blank application will accompany the Annual Circular. Admission fee, $2.00; annual dues, $5.00, entitling the member to the Annual Volume of Transactions.

PEMBERTON DUDLEY, M. D.,
*General Secretary.*

FIFTEENTH AND MASTER STREETS,
PHILADELPHIA, PA.

